# Two-point immobilization of M3 muscarinic receptor: a method for recognizing receptor antagonists in natural products

**DOI:** 10.1186/s13065-024-01198-z

**Published:** 2024-05-03

**Authors:** Xiaomin Huang, Ting Wang, Ludan Wang, Yantao Sun, Ziru Zhang, Yajun Zhang

**Affiliations:** Key Laboratory of Resource Biology and Biotechnology in Western China, Ministry of Education, Ministry of Life Sciences and Medicine, Northwest University, Xi’an, 710069 China

**Keywords:** Biochromatography, Immobilized M3 muscarinic receptors, BJ-PRO-13a peptide, Drug discovery, Natural products

## Abstract

In the investigation of active ingredients from natural products, current technologies relying on drug–target affinity recognition analysis face significant challenges. This is primarily due to their limited specificity and inability to provide downstream pharmacodynamic information, such as agonistic or antagonistic activity. In this study, a two-point method was developed by immobilizing M3 acetylcholine receptor (M3R) through the combination of the conformation-specific peptide BJ-PRO-13a and the HaloTag trap system. We systematically assessed the specificity of the immobilized M3R using known M3R antagonists (pirenzepine and atropine) and agonists (cevimeline and pilocarpine). By frontal analysis and nonlinear chromatography, the performance of immobilized M3R was evaluated in terms of binding kinetics and thermodynamics of four drugs to the immobilized M3R. Additionally, we successfully identified two M3R antagonists within an extract from Daturae Flos (DF), specifically hyoscyamine and scopolamine. Our findings demonstrate that this immobilization method effectively captures receptor-ligand binding interactions and can discern receptor agonists from antagonists. This innovation enhances the efficiency of receptor chromatography to determine binding-affinity in the development of new drugs, offering promise for the screening and characterization of active compounds, particularly within complex natural products.

**Supplementary Information:**

The online version contains supplementary material available at 10.1186/s13065-024-01198-z.

## Introduction

The role of identifying bioactive compounds and their impacts on disease prevention and control in drug discovery has been fully recognized [1–3]. Despite substantial investments in pharmaceutical research, the approval rate of innovative drugs has seen a decline in recent decades [4, 5]. This decline can be attributed, in large part, to the growing challenges in the development of new drugs, particularly the limited efficiency of current methodologies for screening lead compounds. Consequently, there is an urgent need for the development of more efficient methodologies.

One emerging technology that shows promise in drug discovery is the screening method based on drug–target affinity from natural products [6]. Various modern techniques have been employed to screen bioactive compounds from natural sources, including targeted fishing magnetic separation, cell membrane chromatography, and receptor chromatography [7, 8]. Of these techniques, receptor chromatography stands out as particularly powerful as it integrates the separation capabilities of chromatography with the discrete binding affinity of receptor proteins, offering high specificity and efficiency for the determination of effective natural bioactive compounds [9, 10]. In our previous research, we successfully constructed chromatographic models involving serotonin transporter, endothelin A receptor, and nuclear peroxisome proliferator-activated receptor gamma, resulting in the recognition of bioactive ingredients in Coptidis rhizoma, Gardeniae fructus, and Siwu decoction [11–13].

However, a limitation of existing receptor chromatography methods is their reliance on affinity binding between the receptor and the drug as the sole indicator. This approach does not distinguish between agonistic and antagonistic activities of the compounds binding with the receptor. Consequently, the lack of specificity and predictability at the early screening stage results in increased risks, extended research cycles, and substantial investments in later pharmacodynamic studies. Thus, there is a need for novel approaches to manipulate the binding selectivity of immobilized receptors in chromatography columns with respect to agonistic or antagonistic ligands.

**Fig. 1:**
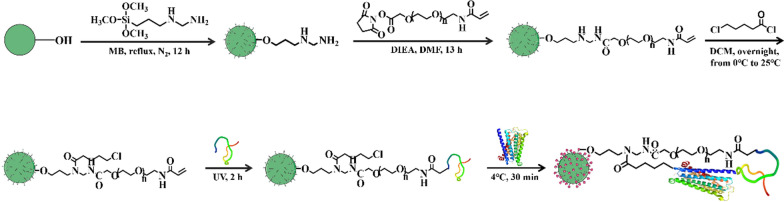
Schematic diagram of M3R immobilization by the two-point method. BJ-PRO-13a peptides were attached to silica gel microspheres, and then M3R was tethered onto the microspheres. The halogenation reaction was used to immobilize the Halo-tagged M3R onto the surface of the microspheres that had been modified with 6-chlorohexanoic acid

Recent findings indicate that receptors bind to agonists and antagonists with different conformational selectivity [14, 15]. Some studies have shown that certain peptides can stabilize the agonist-selective conformations of G protein-coupled receptors (GPCRs) [16, 17]. Inspired by these insights, we hypothesized that combining peptides with classical affinity tags could effectively immobilize GPCRs with capacity to recognize agonists and antagonists. To test this hypothesis, we chose M3 muscarinic receptor (M3R) as our target and used BJ-PRO-13a, a 14-amino acid peptide, to interact with M3R [18, 19]. As illustrated in Fig. 1, we devised a two-point approach for immobilizing the receptor in a specific conformation. This process involved tethering M3R to silica gel microspheres by BJ-PRO-13a and fixing the halo-tagged M3R on the gel surface by a 6-chlorohexanoic acid-mediated halogenation reaction. Our results suggest that this two-point immobilization approach is capable of encapsulating proteins onto solid surfaces in a manner that preserves their orientation and conformation. This method holds promise for enhancing the efficiency of screening for receptor agonists and antagonists in natural products.

## Materials and methods

### Chemicals and instruments

The silica gel microspheres utilized in this study (particle diameter: 7.0 μm; pore size: 250 Å) were a product of the Lanzhou Institute of Chemical Physics, Chinese Academy of Sciences. We procured pirenzepine (CAS: 28797-61-7), atropine (CAS: 51-55-8), darifenacin (CAS: 133099-04-4), cevimeline (CAS: 107233-08-9), and solifenacin (CAS: 242478-37-1) from the Aladdin Industrial Corporation (Shanghai, China). Additionally, pilocarpine (CAS: 92-13-7), acetylcholine (CAS: 51-84-3), and sodium nitrite (CAS: 7632-00-0) were purchased from Macklin Biochemical Co., Ltd. (Shanghai, China). All other chemical reagents were of analytical purity, unless otherwise specified.

The protein marker, ranging from 14.4 kDa to 116 kDa, was provided by Beyotime Biotechnology Co., Ltd. (Shanghai, China). The synthesis of BJ-PRO-13a (with the amino-acid sequence CEGGWPRPGPEIPP) was carried out by GenScript Biotech Corporation (Shanghai, China). The polyclonal antibody against M3R (ACM3) was purchased from ImmunoWay Biotechnology Company (Plano, TX, USA). Alexa Fluor 488 was sourced from Yeasen Biotechnology Co., Ltd. (Shanghai, China). Lastly, we obtained Daturae Flos (DF) from Wenzexuan Chinese Herbal Medicine Company.

For our experiments, we employed a ZEISS Sigma 500 scanning electron microscope (SEM) (Oberkochen, Germany). In addition, a VG Scientific ESCALAB220i-XL analyzer (supplied by Thermo Scientific, Surrey, UK) was used for X-ray photoelectron spectroscopy (XPS). Our chromatographic system comprised an ultraviolet detector, a binary pump, and a column thermostat, belonging to the Elite 3100 series high-performance liquid chromatography (HPLC) instrument made by Dalian Elite Analytical Instrument Company (Dalian, China). The isolation and screening of potential bioactive ingredients were conducted using an Agilent 1290 ion-trap mass spectrometer (Waldbronn, Germany), containing an electrospray ionization interface and a 1290 series binary pump. Additionally, a column oven was utilized for this purpose. Finally, we employed an Eppendorf 5804R refrigerating centrifuge (Hamburg, Germany) for preparation of the cell lysates.

### Establishment of Halo-tagged M3R

We obtained the custom-synthesized HaloTag (Genbank accession: ADN27525.1) from Sangon Biotech (Shanghai, China). A known method was used to express the Halo-tagged M3R in *Escherichia coli* BL21 (DE3) [20]. In brief, we selected a single positive colony and incubated it in 50 mL of Luria–Bertani medium containing ampicillin (100.0 mg/mL) at 37 °C. After 15 h, the growing bacteria were transferred into an auto-induction medium for an additional 12-h incubation. The bacteria were collected, and the pellets were harvested by centrifugation, resuspended in analysis buffer, and disrupted using an Ultrasonic Cell Disruptor. The resulting supernatant was obtained through centrifugation and reserved for the subsequent immobilization steps.

### Two-point immobilization of M3R via the HaloTag and the BJ-PRO-13a peptide

As shown in Fig. 1, the initial process for immobilizing M3R was the modificiton of bare gel using γ-aminoethyl aminopropyltrimethoxysilane. The Schiff base method was utilized to determine the amino content on the silica gel microspheres, revealing a concentration of 194.8 μmol/g. Subsequently, we immersed 1.0 g of the resulting microspheres in 3.0 mL of *N,N*-dimethylformamide (DMF) and reacted them with 246.0 mg of acrylamide PEG2000-*N*-hydroxysuccinate imide ester (ACA-PEG2000-SCM, 1.2 equiv.) and 140.0 μL of *N,N*-diisopropyl ethylamine (4.0 equiv.) for 13 h while stirring at room temperature. After this step, we collected the silica gel microspheres and washed them with DMF three times.

In the next phase, the previously prepared microspheres were immersed in CH_2_Cl_2_ containing EDTA (4.0 equiv.) and reacted with 100.0 μL of 5-chlorovaleryl chloride (6.0 equiv.) by stirring the mixture at room temperature overnight. The microspheres were collected by filtration and then washed with CH_2_Cl_2_, methanol, and ultrapure water, sequentially. Subsequently, we suspended the microspheres in phosphate buffer (20 mM, pH 7.4) to induce the reaction between the BJ-PRO-13a peptide and the microspheres using tris(2-carboxyethyl)phosphine as the mercaptan reductant, which was initiated by ultraviolet radiation (125 W, 365 nm).

Finally, we collected the resulting microspheres by filtration and suspended them in 80 mL of cell lysate containing M3R while stirring for 60 min in an ice bath. Afterward, we separated the prepared microspheres by filtration. These microspheres were rinsed with phosphate buffer (20 mM, pH 7.4), packed into a stainless-steel column (4.6 mm × 30 mm) under a pressure of 400 bar, and stored at 4 °C for further processing.

### Morphological characterization

Morphological analysis of the immobilized M3R was performed by scanning electron microscopy (SEM), X-ray photoelectron spectroscopy (XPS), and immunofluorescence imaging. SEM analysis was carried out on a Hitachi SU8000 field-emission scanning electron microscope equipped with lower and upper secondary electron detectors, a field emission gun, and a Genesis software system. We dispersed the immobilized receptor on double-sided tape of a sample holder which was placed on a cold stage at −150 °C. Observations were conducted when the accelerating voltage and the working distance were 5.0 kV and 9.5 mm.

Elemental analysis of immobilized M3R by XPS was performed on a Thermo Fisher Esca Lab 250Xi analyzer. A monochromatic Al Kα source (1486.6 eV) was employed with electric current and filament voltage of 10 mA and 14.7 keV. All the readout was recorded with a pass energy of 30 eV and 0.5 mm entrance slit of the spectrometer. Compensation of sample charging was realized by the utilization of a charge neutralizer.

To pursue immunofluorescence imaging of the immobilized M3R, we incubated the gels with diverse treatments into tris-buffered saline containing 1% Tween 20 (TBST) in prior to block them with 5% skimmed milk for 1.0 h. We rinsed the microspheres three times with TBST followed by the incubation with 100 μL anti-muscarinic acetylcholine receptor M3/CHRM3 antibody (Abcam, Cambridge, UK) at 4 °C overnight. Washing the result gel with TBST, we incubated the microspheres with 100 μL Alexa Fluor 594 goat anti-rabbit IgG (Yeasen Biotechnology, Shanghai, China) for 1.0 h. Subsequent fluorescence imaging was performed by placing the gel over glass coverslips after total rinsing of the microspheres by TBST.

### Specificity testing of immobilized M3R

The specificity of the immobilized M3R was evaluated by two approaches, of which the first was the two-point immobilized M3R column to analyze the retention times of various compounds, including pirenzepine and atropine (antagonists of M3R) as well as cevimeline and pilocarpine (agonists of M3R). Due to the lack of specific binding sites to the protein, sodium nitrite was used for the experiment to calculate the void time of the chromatographic system. The second approach was to prepare a single-point immobilized M3R column using the HaloTag strategy, according to a previously established method [21]. We conducted control experiments to compare the retention times of five drugs with those observed using the two-point immobilization method. Specificity was defined as acceptable when the relative standard deviation of the retention time between drugs was less than 5.0%.

The detection wavelengths for various compounds were as follows: sodium nitrite, 254 nm; pirenzepine, 280 nm; atropine, 216 nm; cevimeline, 210 nm; and pilocarpine, 230 nm. The mobile phase consisted of phosphate buffer (20 mM, pH 7.4), the flow rate was 0.2 mL/min, and the injection volume was 10 μL.

### Analysis of the ligand-binding activity by the immobilized M3R

#### Analysis based on injection amount

The analytical method based on the injection amount was originally introduced by Professor Zhao in the context of drug–receptor interactions [22]. This method operates under the assumption that the protein sites binding to the stationary phase surface are uniformly distributed, and longitudinal diffusion is negligible. Under these assumptions, we can investigate Eq. (1), which is used to determine the association of the ligand and the immobilizd protein.

1 $$ \frac{{{\text{k}}^{\prime } {\text{nb}}}}{{1 + {\text{k}}^{\prime } }} = {\text{nt}} - \frac{1}{{{\text{kA}}}} \times {\text{k}}^{\prime } {\text{Vm }}\sqrt {b^{2}  - 4ac}  $$

In this equation, “k” represents the capacity factor, which is calculated using the formula k′ = (t_R_–t_0_)/t_0_ for a solute. Here, “t_R_” denotes the retention time of the drug in the M3R column, while “t_0_” represents the void time of the chromatographic system. The symbol “n_b_” signifies the mole amount of the injected solute, “n_t_” corresponds to the number of binding sites on the M3R column, “K_A_” stands for the association constant of the drug binding to the M3 receptor, and “V_m_” is a known constant representing the system’s pipeline volume.

#### Nonlinear chromatography

In our study, nonlinear chromatography was the method used to evaluate the ligand-binding activity of the immobilized M3R [23]. This method relies on the underlying principle that the dynamic interactions between the solute and the immobilized protein govern the shape of the solute’s peak profile. Based on this principle, we calculated both the kinetic and thermodynamic parameters to describe the interactions between the drug and receptor using Eqs. (2) and (3) [24, 25] simultaneously:

2 $$ {\text{y}} = \frac{{{\text{a0}}}}{{{\text{a3}}}}\left[ {{1} - {\text{exp}}\left( { - \frac{{{\text{a3}}}}{{{\text{a2}}}}} \right)} \right]\left[ {\frac{{\sqrt {\frac{{{\text{a1}}}}{{\text{x}}}{\text{I1}}} \left( {\frac{{{2}\sqrt {{\text{a1x}}} }}{{{\text{a2}}}}} \right){\text{exp}}\left( {\frac{{ - {\text{x}} - {\text{a1}}}}{{{\text{a2}}}}} \right)}}{{{1} - {\text{T}}\left( {\frac{{{\text{a1}}}}{{{\text{a2}}}}{,}\frac{{\text{x}}}{{{\text{a2}}}}} \right)\left[ {{1} - {\text{exp}}\left( { - \frac{{{\text{a3}}}}{{{\text{a2}}}}} \right)} \right]}}} \right] $$

3 $$ {\text{T}}\left( {\text{u,v}} \right) = {\text{exp}}\left( { - {\text{v}}} \right)\int\limits_{{0}}^{{\text{u}}} {{\text{exp}}\left( { - {\text{t}}} \right)} {\text{ I0 }}\left( {{2}\sqrt {{\text{vt}}} } \right){\text{dt}} $$

where “x” is the reduced retention time, “y” is the signal intensity, “I_0_” and “I_1_” are the modified Bessel functions, and “T(u, v)” is a switching function used to generate the peak skew. The parameters “a_0_,” “a_1_,” “a_2_,” and “a_3_” correspond to the area, center, width, and distortion, respectively, while “C_0_” represents the concentration of the injected solute multiplied by the width of the injection pulse. The adsorption/desorption rate constants (k_a_/k_d_) and the association constant (K_A_) were calculated by the following equations: k_d_ = 1/a_2_t_0_; k_a_ = k_d_ × K_A_; K_A_ = a_3_/C_0_, which have the same parameters as mentioned above. It is worth emphasizing that the normalized overload parameter (a_3_) should maintain a constant value, whereas the rate parameter (a_2_) and the thermodynamic k′ (a_1_), which can be used to derive binding site densities, should remain unaffected by changes in the concentration.

In this research, we examined the binding interactions of four drugs (atropine, pirenzepine, darifenacin, and solifenacin) with the immobilized M3R through analysis of the injection volume dependence and nonlinear chromatography. Each of the ligand drugs was introduced into the M3R column at varying concentrations: atropine at 0.25, 0.5, 0.75, 1,0, 1.25, 1.5, 2.0, 2.5 and 3.0 mM; darifenacin at 0.1, 0.3, 0.5, 0.7, 1.0, 1.25 and 1.5 mM; pirenzepine 0.01, 0.02, 0.04, 0.08, 0.15, 0.3, 0.6, 1.2, and 1.8 mM; solifenacin at 0.2, 0.4, 0.5, 0.6, 0.8, 0.9 and 1.0 mM. The experimental setup employed a mobile phase of phosphate buffer (20 mM, pH = 7.4), a flow rate of 0.2 mL/min, and an injection volume of 10 μL. For each drug concentration, we performed measurements in triplicate and recorded the resulting elution profiles, followed by their analysis using Peak fit 4.12.

### Identification of receptor antagonists in DF by the immobilized M3R

#### Preparation of the DF extract

**Fig. 2:**
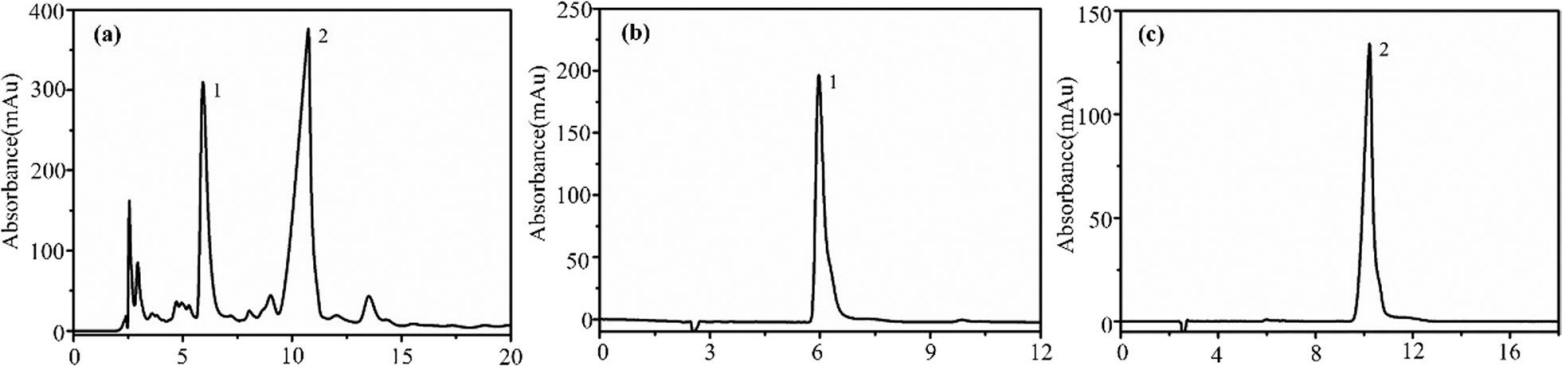
Representative chromatograms of the Daturae Flos extract obtained on a reverse-phase column with a detection wavelength of 216 nm. Reference standards of atropine sulfate (**a**) and (-)-scopolamine hydrochloride (**b**); Daturae Flos extract (**c**). 1, atropine sulfate; 2, (-)-scopolamine hydrochloride

We extended the application of the immobilized M3R to screen for antagonists in a natural product, specifically DF, extracted from the dried flower of *Datura metel* L. To extract the active components, we employed the heating reflux method. In brief, we immersed 10 g of the previously mentioned dried flowers in 100 mL of water for 30 min, followed by two rounds of boiling for 1 h. After filtration, we concentrated the extract to 10 mL by employing rotary evaporation at 60 °C under reduced pressure. To further refine the extract, we subjected it to alcohol sedimentation using a solution of 95% ethanol, resulting in an alcohol content of 75%. Subsequently, we concentrated the liquid supernatant at 60 °C to attain an herbal solution with a concentration of 1.0 g/mL. As illustrated in Fig. 2, the concentrations of scopolamine and atropine in the extracted solution were reduced to 0.22% and 0.08% of the levels present in the raw herb, following the procedures outlined in the Chinese Pharmacopoeia (Pharmacopoeia of PR China, 2020).

### Screening M3R antagonists in the DF extract

To screen for receptor antagonists in the DF extract, we utilized the immobilized M3R column in conjunction with an Agilent 1100 chromatographic system. As part of our control experiments, we also conducted tests using a single-point immobilized M3R column and a mixed sample containing the DF extract and pilocarpine (an M3R agonist) to evaluate the specificity of the immobilized M3R. In these experiments, the mobile phase consisted of 20 mM ammonium acetate buffer, the flow rate was 0.2 mL/min, the injection volume was 10 μL, and the detection wavelength was set at 254 nm. Chromatographic peaks with retention times surpassing the void time were collected and considered as potential M3R antagonists. These fractions underwent further analysis utilizing HPLC/mass spectrometry (MS).

## Results and discussion

### Expression of Halo-tagged M3R

**Fig. 3:**
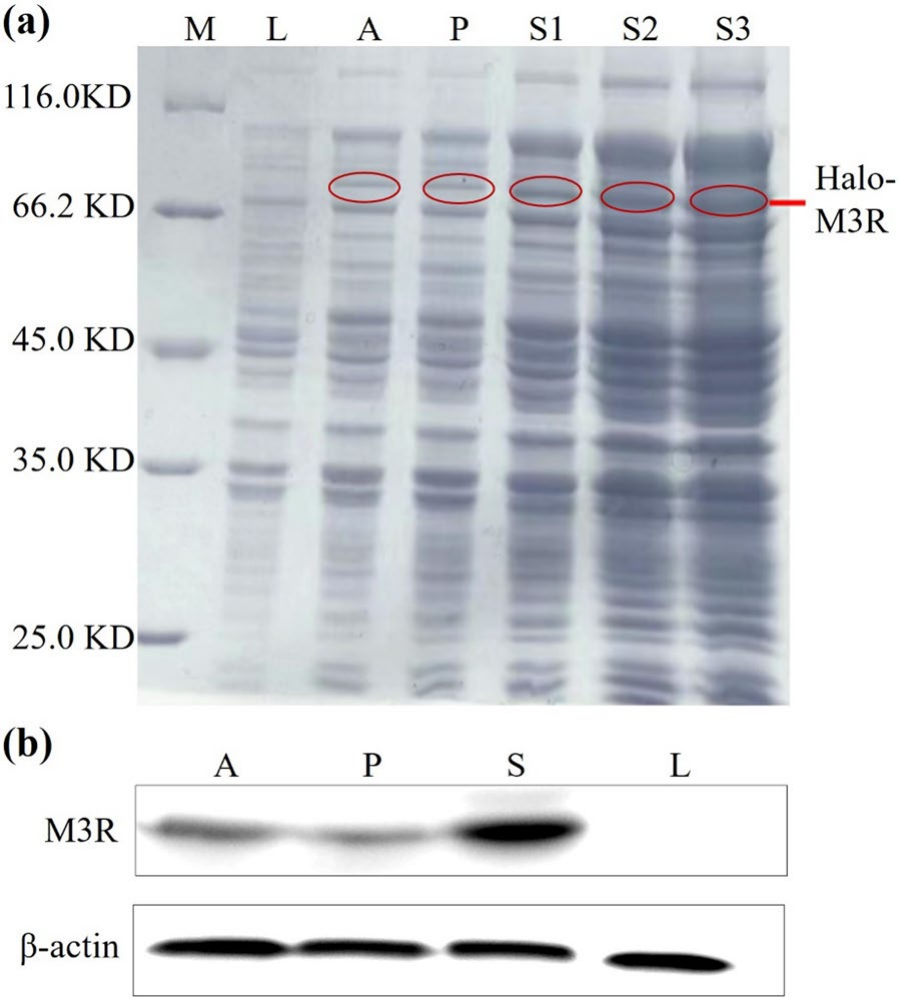
Identification of Halo-tagged M3R in *Escherichia coli*. **a** SDS-PAGE analysis of cell lysates. The circles indicate the location of the M3R; (**b**) Western blot analysis of M3R. Protein marker (**M**), 10 µL of Luria–Bertani medium (**L**), 10 µL of auto-induction medium (**A**), 10, 20, and 30 µL of cell lysate supernatant (S1, S2, S3), and 10 µL of cell lysate precipitate (**P**). Full-length gel and blots are presented in Additional file 1: Fig. S1

To assess the expression of M3R in *E. coli* lysates, we performed sodium dodecyl sulfate–polyacrylamide gel electrophoresis (SDS-PAGE). As depicted in Fig. 3a, a distinct band was clearly observable within the molecular weight range of 66.2–116.0 kDa when autoinduction media was employed. However, this band was conspicuously absent in the negative control lysate (Luria–Bertani medium). Considering the known molecular weights of the HaloTag (33.0 kDa) and M3R (40.4 kDa), we confidently identified this band as the expressed M3R with the HaloTag. Notably, with an increase in the sample injection volume from 10 μL to 30 μL, the band’s intensity progressively increased. Such identification was further confirmed by western blot analysis. As illustrated in Fig. 3b, clear expression of M3R was observed in the cell lysates (A), the supernatants (S), and the precipitates (P). Taking together, we reasoned that the *E. coli* system enabled the expression of Halo-M3R as a functional form when auto-induction media was applied.

### Morphological characterization and elemental analysis of immobilized M3R

**Fig. 4:**
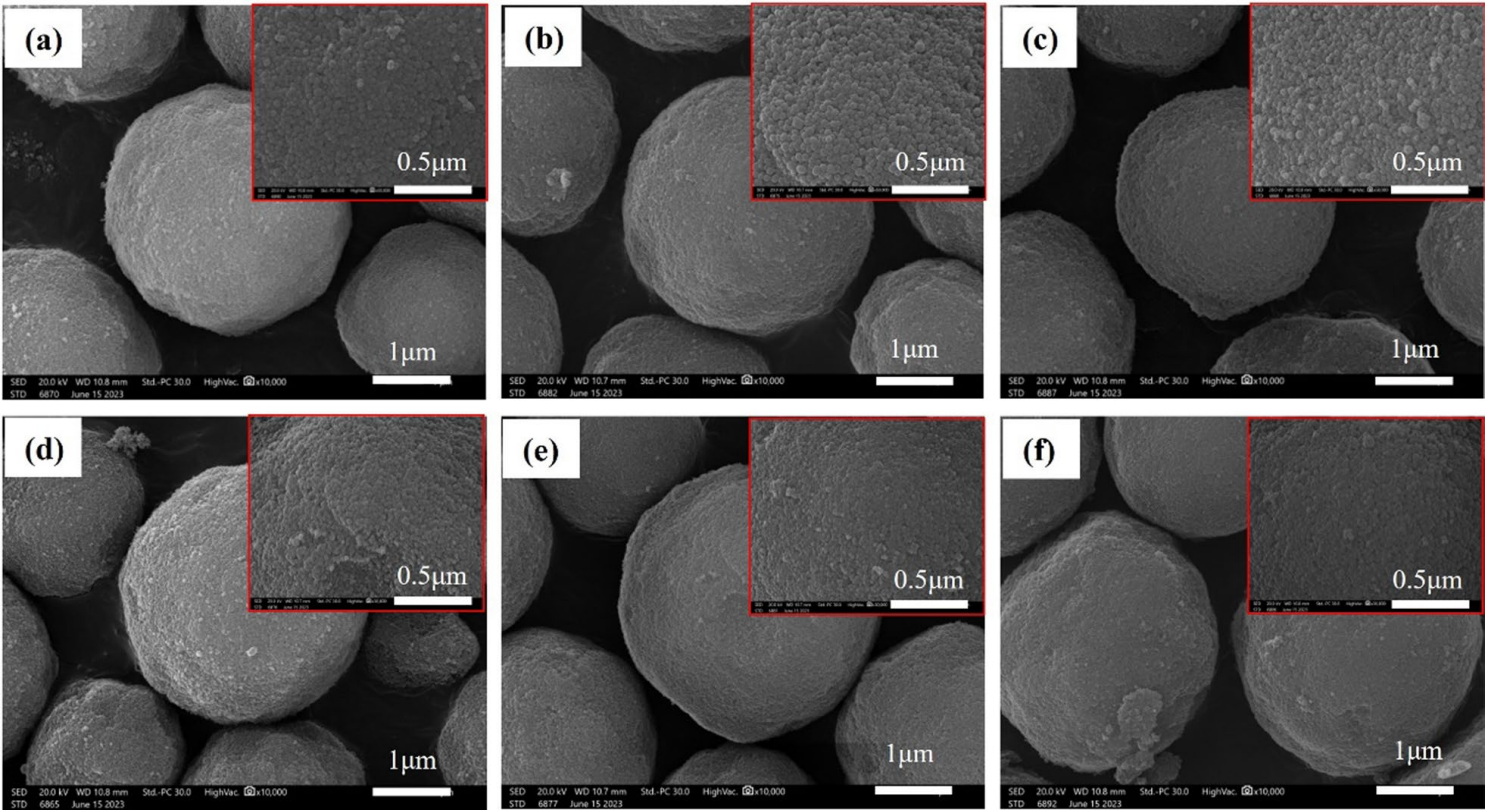
Morphological analysis of microspheres by scanning electron microscopy. Blank silica gel microspheres **a**; peptide-modified microspheres **b**; M3R-coated microspheres **c**; microspheres incubated with *N,N*-dimethylformamide **d**; microspheres incubated with CH_2_Cl_2_ containing EDTA **e**; microspheres incubated with phosphate buffer (20 mM, pH 7.4) (**f**)

We performed a morphological characterization of the immobilized M3R using a SEM. In Fig. 4a–c, we present the SEM images of representative samples: blank silica gel microspheres, peptide-modified microspheres, and M3R-coated microspheres. Initially, the surface of the silica gel microspheres appeared uniform and smooth. However, after peptide modification, the microsphere surface became noticeably rougher and uneven, particularly upon the introduction of M3R. This led us to attribute these clusters to the immobilized receptors.

An additional control was proceeded by incubating the blank silica gel microspheres with the solvents identical to that involved in the synthesized produce. As illustrated in Fig. 4d–f, we found little changes in the morphology between the microspheres incubated with *N,N*-dimethylformamide, CH_2_Cl_2_ containing EDTA, and phosphate buffer (20 mM, pH 7.4). These indicated that little swelling of the microspheres occurred under the proposed reacting conditions.

**Fig. 5:**
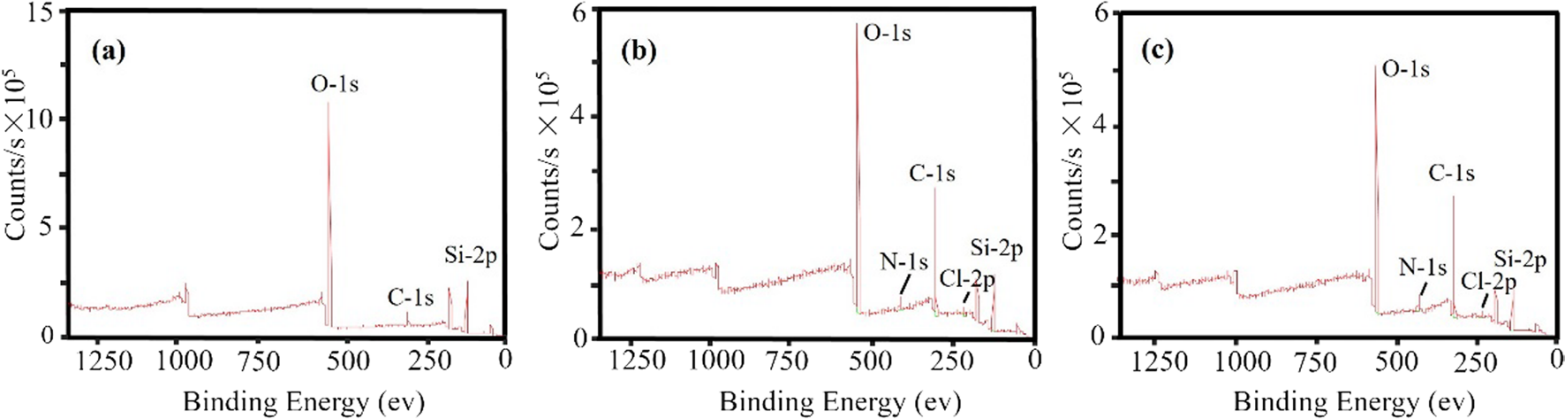
Surface elemental analysis of microspheres by X-ray photoelectron spectroscopy**.** Blank silica gel microspheres (**a**); peptide-modified microspheres (**b**); M3R-coated microspheres (**c**)

To verify whether M3R was successfully affixed to the microspheres, we employed XPS analysis as a probing technique to examine elemental alterations. The results, depicted in Fig. 5 and summarized in Table 1, reveal distinct spectral patterns. The blank silica gel microspheres exhibited peaks at 532 eV, 285 eV, and 103 eV, corresponding to O-1 s, C-1 s, and Si-2p, respectively (Fig. 5a). In comparison, the relative concentrations of the elements O and Si were decreased on the surface of the peptide-modified microspheres. The successful linkage to the peptide was evident as a distinct N-1 s peak at 400 eV (Fig. 5b). Following the binding of M3R, an increase in amino and carboxylic groups within M3R led to higher relative concentrations of the elements N and C and a decrease in the amount of the element O (Fig. 5c). These findings confirm the successful bonding of M3R to the microspheres.

**Table 1:** The binding energy and relative contents of C, N, O, Cl, and Si on the surfaces of blank silica gel microspheres, peptide-modified microspheres, and M3R-coated microspheres

	Position (eV)			Atomic/%		
	Blank silica gel microspheres	Peptide-modified microspheres	M3R-coated microspheres	Blank silica gel microspheres	Peptide-modified microspheres	M3R-coated microspheres
C-1 s	284.82	285.43	285.35	11.54	47.19	49.97
N-1 s	–	400.05	400.01	–	2.97	4.24
O-1 s	532.86	532.34	532.28	57.27	33.30	31.19
Cl-2p	–	200.61	200.31	–	1.84	1.17
Si-2p	103.27	103.23	103.17	31.19	14.70	13.43

**Fig. 6:**
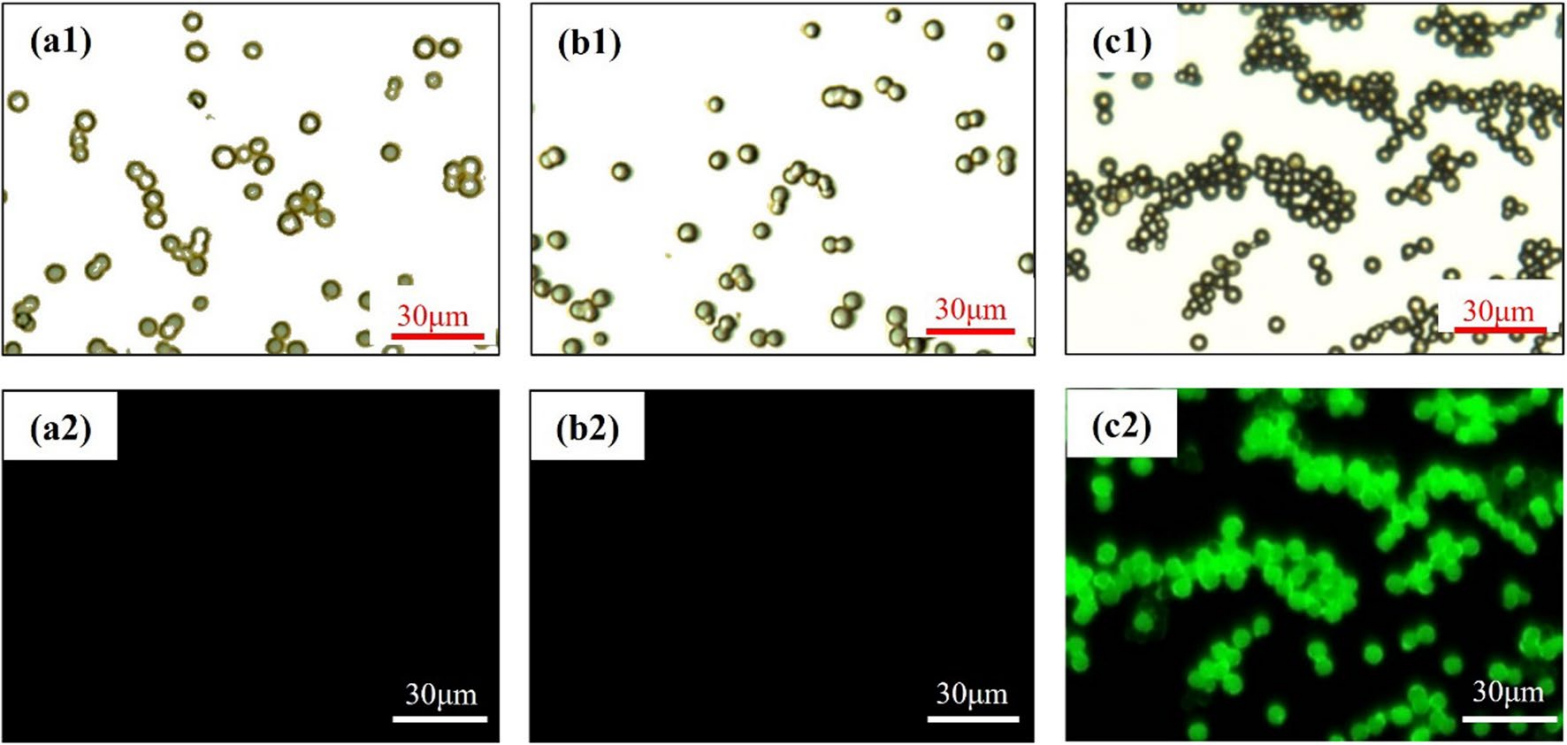
Characterization of the microspheres by immunofluorescence analysis**.** White light images of the microspheres (**a1–c1**) and fluorescence images of the microspheres (**a2–c2**). Blank silica gel microspheres (**a**); peptide-modified microspheres (**b**); M3R-coated microspheres (**c**)

To validate the presence of M3R on the microspheres, we conducted an immunofluorescence analysis. Following the kit protocol, we incubated blank silica gel microspheres, peptide-modified microspheres, and M3R-coated microspheres with the ACM3 polyclonal antibody and then introduced Alexa Fluor 488 for image development. Figure 6 displays the images of these three types of microspheres captured with a fluorescence microscope. Under white light, all three types of microspheres exhibited the same spherical morphology (Fig. 6a1–c1). However, when excited with a fluorescence wavelength of 450–490 nm, the blank silica gel microspheres and peptide-modified microspheres displayed no fluorescence (Fig. 6a2, b2), whereas the M3R-coated microspheres exhibited strong green spherical fluorescence (Fig. 6c2). We attribute these differences to the immobilization of M3R on the microspheres, which allows them to bind with the ACM3 polyclonal antibody and generate the observed fluorescence.

To determine the quantity of BJ-PRO-13a immobilized on the silica gel microspheres, we employed the bicinchoninic acid assay. We found that 1 g of microspheres contained 906.1 ± 3.9 μg of BJ-PRO-13a. As a control, we also determined the peptide content adsorbed on silica gel microspheres, which amounted to 15 ± 0.3 μg of peptide per g of microspheres. This stark contrast in peptide content between the peptide-modified microspheres and the control demonstrates the successful fixation of BJ-PRO-13a onto the microspheres.

### Specificity of the immobilized M3R to recognize receptor antagonists

**Fig. 7:**
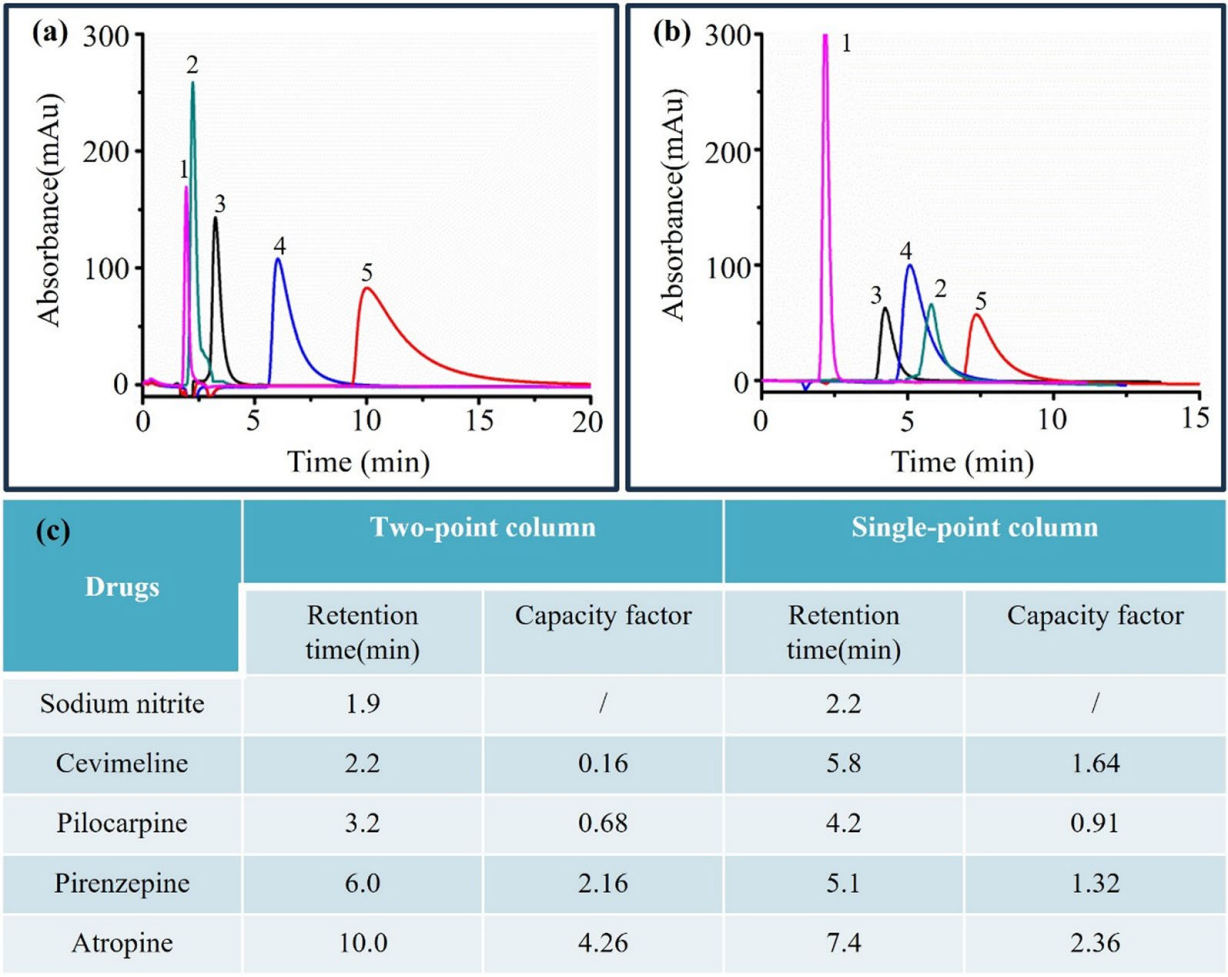
Specificity characterization of the immobilized M3R. The chromatograms of drugs on a two-point column (**a**) and a single-point column (**b**), and the retention times of drugs (**c**). 1, sodium nitrite; 2, cevimeline; 3, pilocarpine; 4, pirenzepine; 5, atropine

Figure 7a presents the chromatographic retention profiles of sodium nitrite, cevimeline, pilocarpine, pirenzepine, and atropine on the two-point immobilized M3R column. At a flow rate of 0.2 mL/min, we established a reference retention time of 1.9 min for sodium nitrite, which also served as a reference point for the chromatographic system. Similarly, atropine, pirenzepine, cevimeline, and pilocarpine exhibited retention times of 10.0 min, 6.0 min, 2.2 min, and 3.2 min, respectively. Notably, we observed robust retention of the two antagonists, pirenzepine and atropine, on the M3R column; whereas there was relatively weak retention of cevimeline and pilocarpine, which are agonists of M3R (Fig. 7a, c). In contrast, when the same compounds were analyzed on the single-point immobilized M3R column, their retention times shifted to 7.4 min for atropine, 5.1 min for pirenzepine, 5.8 min for cevimeline, and 4.2 min for pilocarpine (Fig. 7b, c). These results confirm that the two-point immobilized M3R exhibits conformation-specific behavior, retaining its original binding preference for antagonists, while the single-point immobilized M3R interacts effectively with both antagonists and agonists.

It is worthy of stating that it is not an issue even though there is a significant lack of separation between the various drug peaks. Typically, affinity chromatography like this work is clear different from HPLC, in particularly when it serves as a screening method. In classical HPLC method, we pursue the separation capacity of the column, while in affinity chromatographic technique, we intend to focus on the specificity of the column to recognize binders from co-existing contaminations. With an exception of sodium nitrite, all the canonical drugs that has proved to interact with M3R exhibit retentions longer than the void time on the single-point immobilized receptor. This indicated that the column has capacity to bind to the receptor ligands. As anticipated, the retention of agonists (cevimeline, pilocarpine) clearly reduced when two-point immobilized receptor is applied. Conversely, the antagonists (pirenzepine, atropine) exhibited enhanced retentions in comparison with the single-point column. These demonstrated a conformation-selective affinity of the two-point column to the receptor ligands, upon which we foresee the distinguishment of M3R antagonists from the receptor agonists. As such, the two-point column is capable of recognizing M3R antagonists from complex sample like natural products as non-binders will exhibit retentions approximated to the void time. To summarize, the separation is not a case in affinity chromatographic screening method since all the peaks with good retentions are generated by the high specificity of the column.

In this study, we observed a loss of agonist retentions and an enhancement of antagonist bindings when peptide BJ-PRO-13a was introduced into M3R immobilization. This indicated that the use of the peptide changed the specificity of the receptor in terms of its affinity to the ligands. As the nature of affinity is the forces including hydrogen bond, electrostatic interaction, and Van der Waals, it is determined by stereostructure of ligand binding amino acids and their distance and positions. These are often determined by the conformation of receptor. As such, we deduced that the presence of the peptide BJ-PRO-13a altered the binding sites of the four ligands through changing the receptor conformation. This may elucidate the varying retention behaviors of antagonists and agonists observed on the two-point column.

### Stability of the immobilized M3R

**Fig. 8:**
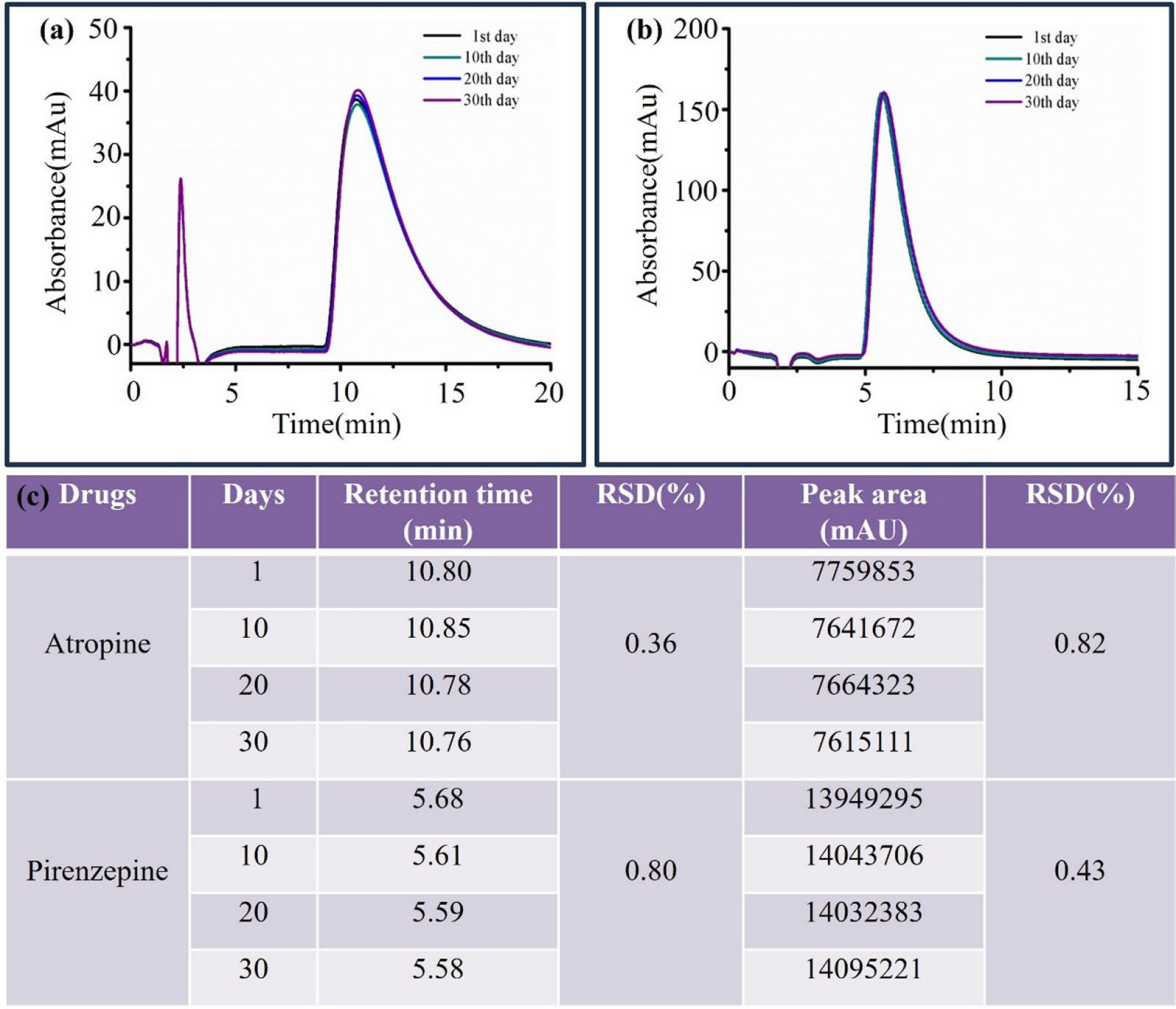
Stability of the M3R column over thirty days. The chromatograms of atropine (**a**) and pirenzepine (**b**), and the retention times and peak areas of atropine and pirenzepine (**c**)

To assess the stability of the immobilized M3R, we analyzed the peak areas and retention times for atropine and pirenzepine over a span of 0, 10, 20, and 30 days. In this case, all the drug samples were stored at 4.0 °C according to manufacture instructions, under which the drugs have reported to remain stable without demonstrable degradation over at least 30 days. As illustrated in Fig. 8, both atropine and pirenzepine exhibited consistent peak profiles and retention times, with standard deviations consistently measuring less than 5.0%. These results offer compelling evidence of the immobilized M3R’s stability and ability to accurately detect ligand retention times over an extended period of roughly 30 days. This extended stability duration provides ample time for investigating drug–receptor interactions and lead compounds within natural products.

### Ligand-binding activity of the M3R column

**Fig. 9:**
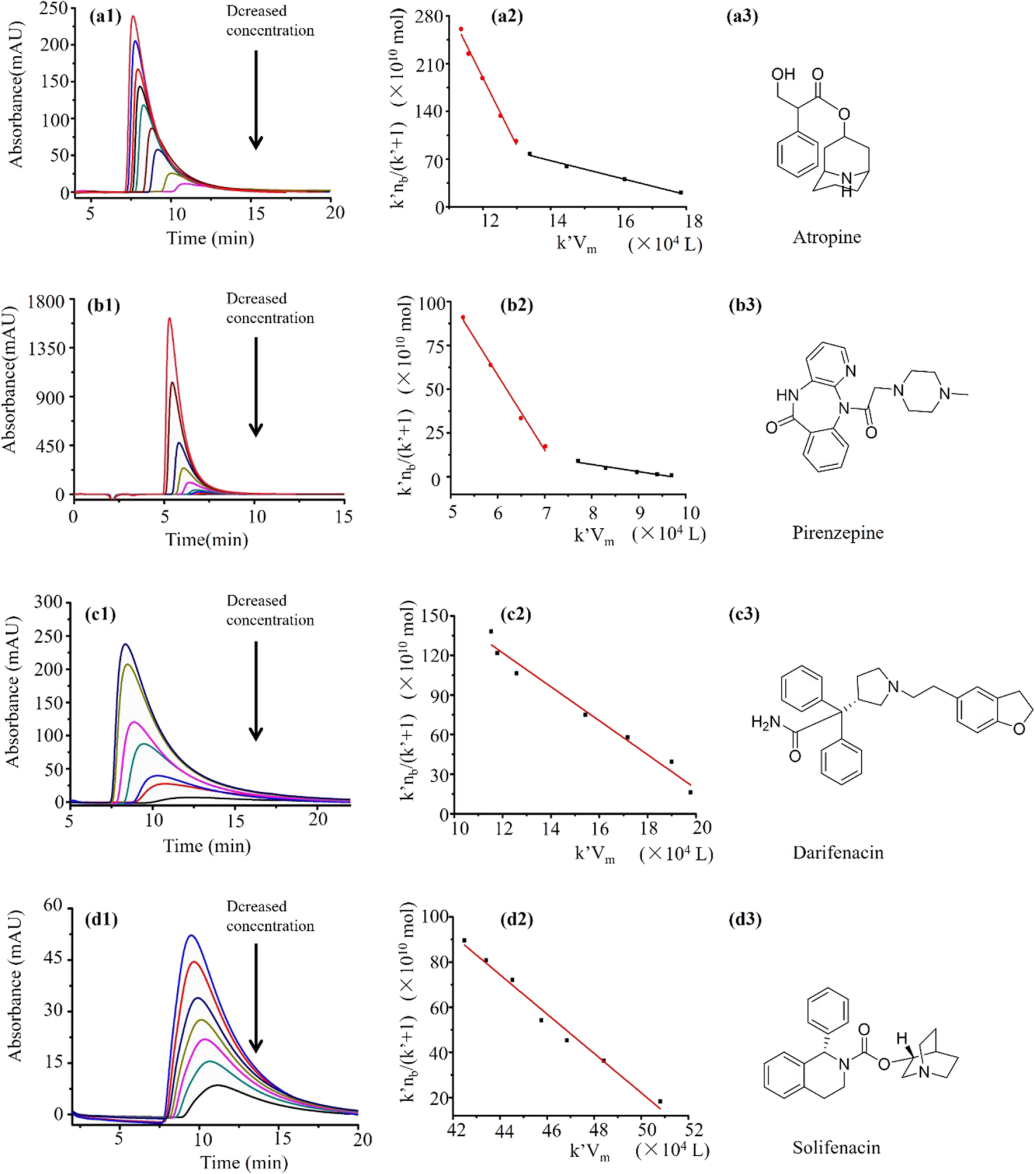
The interactions of the four drugs with M3R as determined using the injection amount-dependent method. The chromatograms of atropine (**a1**), pirenzepine (**b1**), darifenacin (**c1**), and solifenacin (**d1**) were obtained using the M3R column, and the ligand concentration decreased from top to bottom. Based on the curve of k′n_b_/(1 + k′) and k′V_m_, we obtained the regression curves of atropine (**a2**), pirenzepine (**b2**), darifenacin (**c2**), and solifenacin (**d2**). Here, a3, b3, c3, and d3 are the structural formulas of atropine, pirenzepine, darifenacin, and solifenacin, respectively

In our study, we employed an injection amount-dependent analytical method, utilizing varying concentrations of atropine, pirenzepine, darifenacin, and solifenacin. As depicted in Fig. 9a1–d1, with increasing injection amounts of the ligand drugs, there was a decrease in the retention times for atropine, pirenzepine, darifenacin, and solifenacin. Simultaneously, their capacity factor (k′) displayed a consistent pattern across the spectrum of drug concentrations. These observations validate the suitability of employing Eq. (1) for the analysis of binding interactions between the immobilized M3R and the four drugs. Figure 9a2–d2 illustrates the typical plots of k′n_b_/(1 + k′) against k′V_m_ for the four drugs. Notably, we observed two linear relationships for atropine and pirenzepine, indicating the presence of two distinct binding site types. In contrast, darifenacin and solifenacin exhibited a robust linear relationship within the range of injection concentrations, indicating the presence of a single type of binding site between the immobilized M3R and these two drugs.

As shown in Table 2, our results revealed the association constants (K_A_) for atropine, which were (8.00 ± 0.20) × 10^4^ M^−1^ and (0.98 ± 0.02) × 10^4^ M^−1^ at concentrations less than and greater than 1 mM, respectively. Additionally, the numbers of binding sites (n_t_) were 2.42 ± 0.60 nM and 141.00 ± 2.83 nM, respectively. Similarly, pirenzepine exhibited association constants (K_A_) of (26.04 ± 0.82) × 10^4^ M^−1^ and (2.34 ± 0.07) × 10^4^ M^−1^ at concentrations less than and greater than 0.15 mM, respectively, with the corresponding numbers of binding sites (n_t_) being 3.75 ± 0.12 nM and 31.40 ± 0.87 nM, respectively. For darifenacin and solifenacin, the association constants (K_A_) were (7.75 ± 0.20) × 10^4^ M^−1^ and (12.01 ± 0.08) × 10^4^ M^−1^, respectively, while the numbers of binding sites (n_t_) were 27.60 ± 0.35 nM and 44.00 ± 0.26 nM, respectively. Importantly, all correlation coefficients for the four drugs exceeded 0.98, underscoring the robustness of our findings.

**Table 2:** The constants of atropine, pirenzepine, darifenacin, and solifenacin with the immobilized M3R by the injection volume-dependent method

Drug	Concentration (μM)	k′n_b_/(1 + k′) = n_t_–(1/K_A_)k′Vm	R^2^	K_A_ (10^4^ L/mol)	n_t_ (× 10^−9^ mol/L)
Atropine	250–1000	k′n_b_/(1 + k′) = 2.42 × 10^–8^–1.25 × 10^–5^ k′Vm	0.993	8.00 ± 0.20	2.42 ± 0.60
	1250–4000	k′n_b_/(1 + k′) = 1.41 × 10^–7^–1.02 × 10^–4^ k′Vm	0.990	0.98 ± 0.02	141.00 ± 2.83
Pirenzepine	10–150	k′n_b_/(1 + k′) = 3.75 × 10^–9^–3.84 × 10^–6^ k′Vm	0.986	26.04 ± 0.82	3.75 ± 0.12
	300–1800	k′n_b_/(1 + k′) = 3.14 × 10^–8^–4.27 × 10^–5^ k′Vm	0.987	2.34 ± 0.07	31.40 ± 0.87
Darifenacin	500–2000	k′n_b_/(1 + k′) = 2.76 × 10^–8^–1.29 × 10^–5^ k′Vm	0.977	7.75 ± 0.20	27.60 ± 0.35
Solifenacin	200–1000	k′n_b_/(1 + k′) = 4.40 × 10^–8^–8.33 × 10^–6^ k′Vm	0.976	12.01 ± 0.08	44.00 ± 0.26

**Fig. 10:**
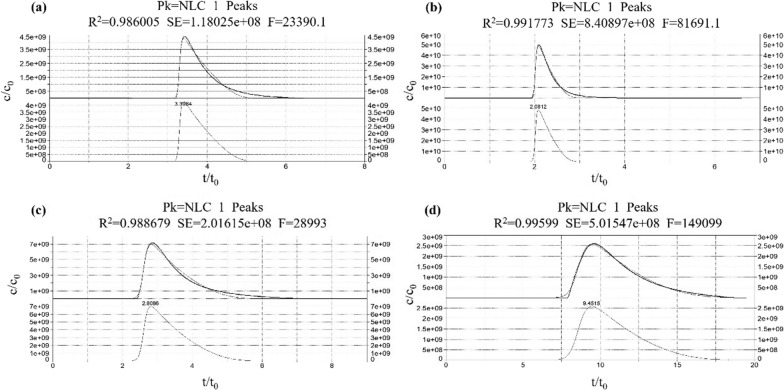
The interactions of the four drugs with M3R by nonlinear chromatography Displayed are the experimental peak profiles for atropine (**a**), pirenzepine (**b**), darifenacin (**c**), and solifenacin (**d**) on the M3R column. The top section shows these profiles with the fitted nonlinear chromatography functions, while the bottom represents the fitted nonlinear chromatography functions in isolation. Injection concentration: 0.4 mM; injection volume: 10 μL. The Y-axis has been scaled to y = Y/C_0_, while the X-axis, representing the retention time (X), has been scaled to x = X/t_0_.

In our pursuit of understanding receptor–ligand binding kinetics, we introduced nonlinear chromatography as a valuable tool. To exemplify this approach, we present representative chromatograms in Fig. 10, depicting the experimental peaks of the four drugs at an injection concentration of 0.4 mM. Subsequently, we systematically processed the raw data from these recorded peak profiles using Peak Fit 4.12. This involved baseline removal and refitting of the curves to derive precision values (a_0_, a_1_, a_2_, and a_3_) for each concentration of atropine, pirenzepine, darifenacin, and solifenacin. These precision values allowed us to calculate and obtain essential kinetic parameters, namely K_A_, k_d_, and k_a_, as summarized in Table 3. The K_A_ values of atropine were (1.28 ± 0.02) × 10^4^ M^−1^ and (0.20 ± 0.01) × 10^4^ M^−1^, respectively, and the k_d_ values were found to be 63.02 ± 0.01 s^−1^ and 89.94 ± 0.01 s^−1^ at concentrations less than and greater than 1 mM, respectively. Pirenzepine displayed K_A_ values of (6.66 ± 0.17) × 10^4^ M^−1^ and (0.12 ± 0.01) × 10^4^ M^−1^ at concentrations less than and greater than 0.15 mM, respectively, with corresponding k_d_ values of 59.54 ± 0.09 s^−1^ and 133.24 ± 0.01 s^−1^, respectively. For darifenacin and solifenacin, we determined the K_A_ values to be (4.68 ± 0.88) × 10^4^ M^−1^ and (7.28 ± 0.48) × 10^4^ M^−1^, respectively, while the k_d_ values were 30.53 ± 0.88 s^−1^ and 9.14 ± 0.25 s^−1^, respectively.

**Table 3:** The binding parameters of atropine, pirenzepine, darifenacin, and solifenacin with the immobilized M3R by nonlinear chromatography

Drug	Concentration (μM)	K_A_ (× 10^4^ M^−1^)	k_a_ (× 10^6^ s^−1^ M^−1^)	k_d_ (s^−1^)
Atropine	250–1000	1.28 ± 0.02	0.81 ± 0.03	63.02 ± 0.01
	1250–3000	0.20 ± 0.01	0.18 ± 0.01	89.94 ± 0.01
Pirenzepine	10–150	6.66 ± 0.17	3.93 ± 0.07	59.54 ± 0.09
	300–1500	0.12 ± 0.01	0.16 ± 0.01	133.24 ± 0.01
Darifenacin	500–2000	4.68 ± 0.88	1.43 ± 0.25	30.53 ± 0.88
Solifenacin	200–1000	7.28 ± 0.48	0.66 ± 0.04	9.14 ± 0.25

An intriguing observation is that when compared to the injection amount-dependent method, the K_A_ values obtained through nonlinear chromatography remained at similar levels for all four drugs. This consistency underscores the reliability of the nonlinear chromatographic approach for determining these critical kinetic parameters.

### Identifying M3R antagonists in DF

DF, derived from the dried flowers of *D. metel* L., has been applied in traditional Chinese medicine to alleviate smooth muscle spasms and pain. Some bioactive constituents within DF are known to interact with M3R, contributing to its pharmacological effects. Our specificity assessment demonstrated that the M3R column had the capacity to selectively bind receptor antagonists, effectively excluding agonists and other herbal ingredients.

**Fig. 11:**
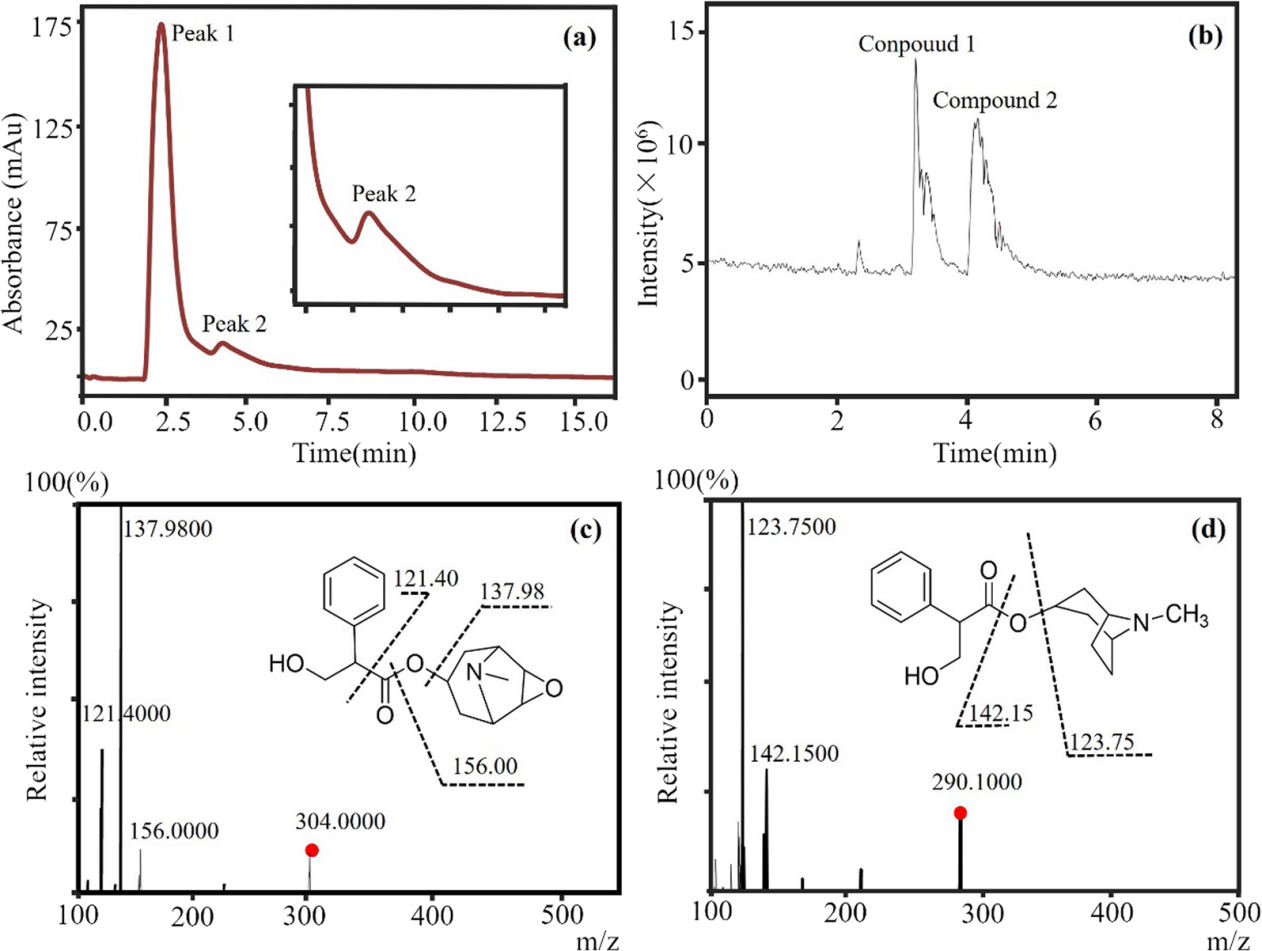
Identification of M3R antagonists from Datura Flos using the immobilized M3R. **a** Shown here is a representative chromatogram of the Daturae Flos extract on the immobilized M3R column. Peak 2, characterized by a retention time exceeding the void time, is indicative of compounds within the extract that specifically interact with M3R. **b** The total ion current corresponding to peak 2 in the chromatogram was measured utilizing a reverse-phase XDB-C_18_ column (2.1 mm × 150 mm, 3.5 μm) in conjunction with an electrospray-MS/MS system. **c** Hyoscyamine was conclusively determined as compound 1 via HPLC–MS/MS analysis. **d** Scopolamine was established as compound 2 through HPLC–MS/MS analysis

As depicted in Fig. 11a, our chromatographic analysis unveiled two distinct peaks. The initial peak, appearing at 2.5 min, was characterized as a general mixture without distinct affinity for the immobilized M3R, as its retention time closely mirrored the column’s void time. In contrast, the second peak at 4.3 min exhibited a retention time that is longer than the void time, suggesting the retention of compounds associated with M3R.

Subsequent HPLC-quadrupole time-of-flight-MS analysis shed light on the composition of this eluted mixture, revealing two primary components with ions at *m/z* 304.00 [M + H]^+^ and 290.10 [M + H]^+^, corresponding to molecular weights of 303.10 and 289.20, respectively (Fig. 11b). Further scrutiny of the daughter ions of *m/z* 304.00 unveiled fragments at 156.00 [M–C_9_H_6_O_2_]^+^, 137.980 [M–C_9_H_6_O_2_–H_2_O]^+^, and 121.40 [M–C_9_H_13_NO_3_]^+^. Similarly, the daughter ions of *m/z* 390.10 included fragments at 142.15 [M–C_9_H_6_O_2_]^+^ and 123.75 [M–C_9_H_6_O_2_–H_2_O]^+^ (Fig. 11c, d). Based on these findings, we confidently identified these compounds as scopolamine and hyoscyamine, both well-known M3R antagonists [26, 27]. This discovery underscores the specificity of the M3R column for recognizing receptor antagonists while excluding agonists and other herbal constituents.

**Fig. 12:**
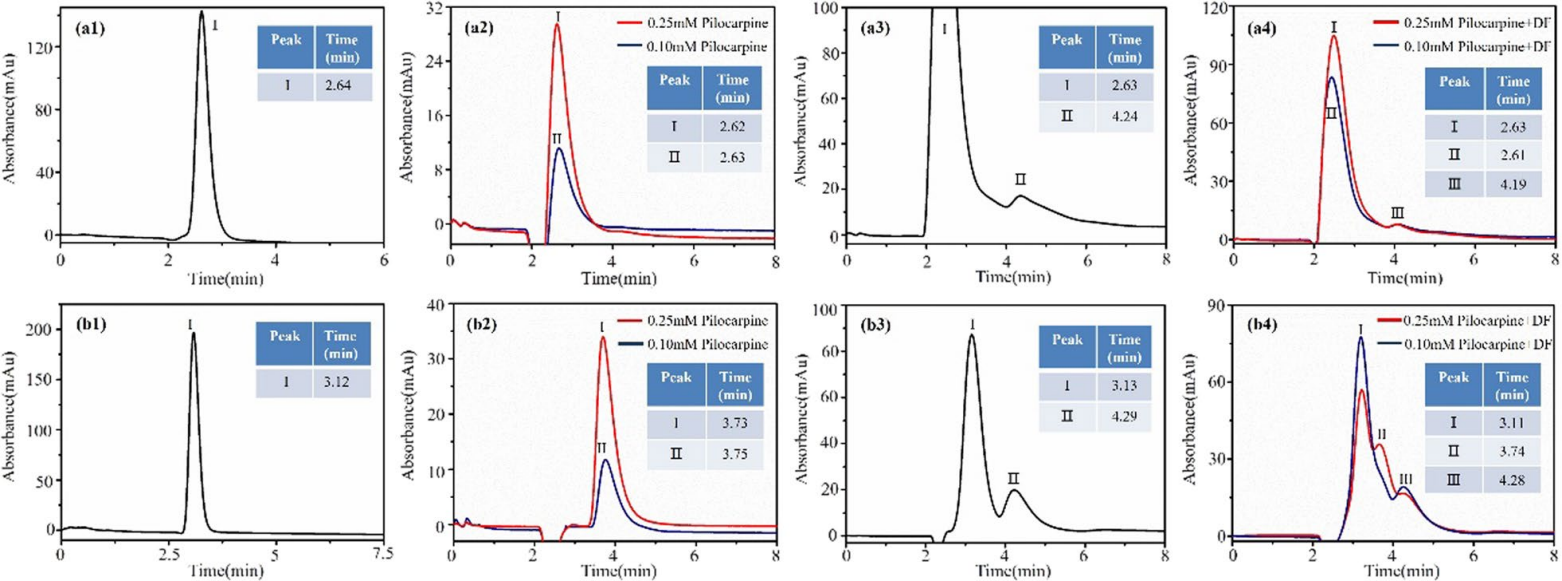
The retention behaviors of a sample mixture of Daturae Flos extract and pilocarpine on the immobilized M3R columns. Two-point immobilized M3R column (**a**) and Single-point immobilized M3R column (**b**). Chromatograms of sodium nitrite (a1, b1), pilocarpine (a2, b2), Daturae Flos extract (c1, c2), and sample mixture (d1, d2)

To validate the capability of the M3R column to distinguish between antagonists and agonists within DF, we prepared a sample mixture containing pilocarpine, a known M3R agonist, and DF extract for analysis. As anticipated, the sample mixture exhibited chromatographic peaks on the M3R column that closely resembled those found in the DF extract. These included peaks at 2.1 min and 4.2 min (Fig. 12a1–a4). Notably, the peak corresponding to pilocarpine was obscured by the presence of other unbound substances present in DF.

In contrast, when utilizing a single-point immobilized M3R column, the peaks of pilocarpine and the antagonists in the sample mixture were clearly resolved at 3.7 min and 4.3 min, respectively, while the retention time for nonspecific substances interacting with M3R was 3.1 min (Fig. 12b1–b4). In the case of the sample mixture containing 0.1 mM pilocarpine, a distinct pilocarpine peak was not readily discernible due to its smaller size and interference from unbound DF components (Fig. 12b4).

These findings provide compelling evidence supporting the feasibility and effectiveness of the two-point immobilization of M3R for identifying M3R antagonists within natural products while distinguishing them from agonists, thereby enhancing the specificity of our analytical approach.

DF is known to harbor a diverse array of compounds, encompassing alkaloids, terpenes, withanolides, flavonoids, and amides [28]. Among these, tropane alkaloids, notably scopolamine, hyoscyamine, anisodine, and anisodamine, are recognized as the principal active components in DF that target M3R [29]. Our research, which identified hyoscyamine and scopolamine as M3R antagonists within DF, aligns seamlessly with these established findings. The mechanisms responsible for the spasmolytic activity of hyoscyamine and scopolamine are rooted in their ability to inhibit M3R in effector cells and to block the transmission of acetylcholine, thereby inducing relaxation in the smooth muscles of bronchial and other tissues. These findings underscore the reliability and accuracy of our method for identifying M3R antagonists within complex mixtures.

It is important to highlight that we did not identify other compounds present in the DF extract, such as anisodamine and anisodine, when utilizing the M3R column. This result might be attributed to either the low affinity of subtype-selective ligands for M3R within our column or the low amounts of these compounds in the sample. Further investigations are warranted to explore the presence of additional M3R agonists and antagonists, contributing to a more comprehensive understanding of the pharmacological profile of DF.

## Conclusion

In this study, a novel method was developed to immobilize M3R utilizing a combination of the HaloTag fusion system and a specific peptide. This approach offers several distinct advantages: (1) Enhanced stability: The immobilized receptors demonstrate remarkable stability due to the dual-point affinities employed in their immobilization. (2) Minimized nonspecific binding: Undesired protein adsorption is significantly reduced as the immobilized receptors exclusively engage in two-point interactions with the target receptor. (3) Precise orientation and conformation: The method enables the precise orientation and conformational immobilization of the receptors, ensuring their functional integrity. (4) Accurate recognition: The immobilized receptors exhibit the unique ability to accurately distinguish between receptor agonists and antagonists. This distinctive capability holds great promise, rendering our method a potential platform for the precise screening of ligands derived from natural plants that target an immobilized receptor. Such applications extend to lead screening, druggability assessments, and clinical diagnosis, highlighting the versatility and potential impact of this innovative approach in the field of pharmacological research and drug development.

### Supplementary Information

**Additional file 1: Fig. S1.** **a** Full-length SDS-PAGE gel of cell lysates from *Escherichia coli* expressing Halo-M3R. **b** Full-length western blot of M3R. **c** Full-length western blot of β-actin. The lines indicate where the gel and blot were cropped.

## Data Availability

Data is provided within the manuscript or Additional file. The amino acid sequence of HaloTag was from the Genbank (www.ncbi.nlm.nih.gov/protein) with accession code ADN27525.
