# Supplementary material for: Two-point immobilization of M3 muscarinic receptor: a method for recognizing receptor antagonists in natural products
Source: BMC Chem. 2024 May 3;18(1):94. doi: 10.1186/s13065-024-01198-z (PMC11069257; doi:10.1186/s13065-024-01198-z)
Supplement: Supplementary file 1 — Additional file 1: Fig. S1. a Full-length SDS-PAGE gel of cell lysates from Escherichia coli expressing Halo-M3R. b Full-length western blot of M3R. c Full-length western blot of β-actin. The lines indicate where the gel and blot were cropped. [file 13065_2024_1198_MOESM1_ESM.docx]

**Fig. S1. (a) Full-length SDS-PAGE gel of cell lysates from *Escherichia coli* expressing Halo-M3R. (b) Full-length western blot of M3R. (c) Full-length western blot of β-actin.** The lines indicate where the gel and blot were cropped.

(a)


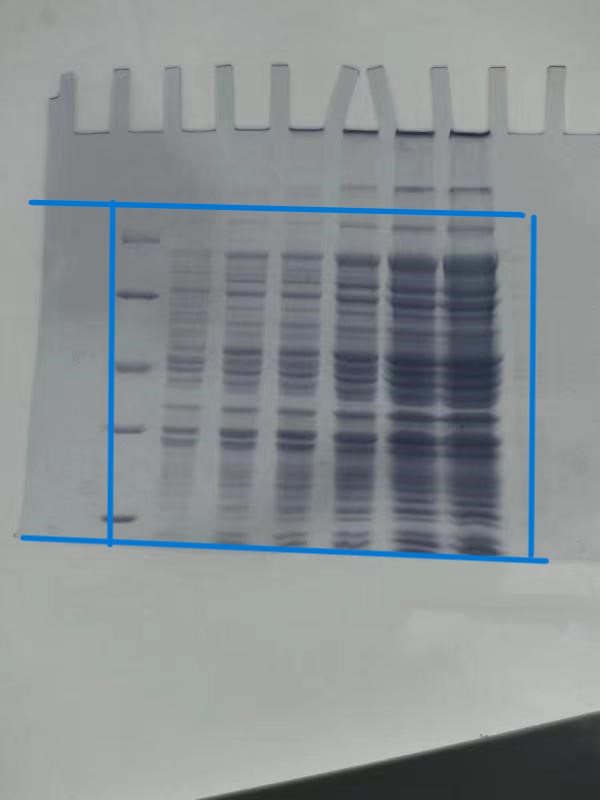


**(b)**

**

**

Marker

**(c)**





Marker
